# Overexpression of EWSR1 (Ewing sarcoma breakpoint region 1/EWS RNA binding protein 1) predicts poor survival in patients with hepatocellular carcinoma

**DOI:** 10.1080/21655979.2021.1982844

**Published:** 2021-10-06

**Authors:** Weijie Jiang, Tao Wu, Xuan Shi, Jiawen Xu

**Affiliations:** a Department of Pathology, The People’s Hospital of Jianyang City, Jianyang, Sichuan, 641499, China; b Department of Pathology, Shandong Provincial Hospital affiliated to Shandong First Medical University, Jinan, Shandong, 250021, China; c Department of Pathology, Shandong Provincial Hospital affiliated to Shandong University, Jinan, Shandong, 250021, China

**Keywords:** Hepatocellular carcinoma, EWSR1, prognostic marker, bioinformatics analysis

## Abstract

Hepatocellular carcinoma (HCC) is one of the most prevalent malignant neoplasms with high relapse and mortality rate. It is of great importance to identify novel and effective molecular markers to predict prognosis for the treatment of HCC. The Ewing sarcoma breakpoint region 1 (EWSR1) gene is well known to fuse with various partner genes and involved in promoting the development of multiple sarcomas, especially the Ewing sarcoma family of tumors. Nevertheless, seldom studies have focused on the role of EWSR1 in cancers of epithelial origin, let alone in HCC. In the current study, the transcriptional and clinical data of EWSR1 in HCC patients were obtained from TCGA and GEO databases, as well as 124 cases from the department of Pathology of Sichuan Jianyang People’s Hospital. Kaplan-Meier and Cox regression analysis were used to assess patient prognosis. EWSR1 mRNA levels were significantly upregulated in HCC tissues than in normal liver tissues (*P* < 0.001). The TCGA database analysis showed upregulation of EWSR1 was associated with histological grade, pathologic T stage and death, in addition to that, the T staging, N staging, TNM staging, Ki67, AFP expression were extremely higher in the EWSR1 over-expression group in our cohort. Univariate and multivariate Cox hazard regression analysis results revealed that EWSR1 was an independent prognostic factor for OS in HCC, and bioinformatics analysis showed RNA splicing process represented the major function and pathway. In conclusion, our data showed EWSR1 could serve as a novel promising prognostic biomarker for HCC patients.

**Abbreviations**: AFP, Alpha-fetoprotein; CCL14, C-C motif chemokine ligand 14; CK19, Cytokeratin 19; CI, coefficient interval; COL1A1, Collagen 1A1; DFS, Disease-free Survival; EWSR1, Ewing Sarcoma breakpoint region 1/EWS RNA binding protein 1; FLI1, Friend leukemia virus integration 1; GEO, Gene Expression Omnibus; GO, Gene Ontology; HCC, Hepatocellular carcinoma; HR, Hazard ratio; KEGG, Kyoto Encyclopedia of Genes and Genomes; mRNA, messenger Ribonucleic Acid; N, nodule; OS, Overall survival; PPI, Protein-Protein Interaction analysis; RNA, Ribonucleic Acid; SD, Standard Deviation; TCGA, The Cancer Genome Atlas; T, tumor; TNM, tumor-nodule-metastasis.

## Introduction

Hepatocellular carcinoma (HCC) is one of the most common malignancies worldwide, with increasing incidence and mortality rates, particularly in China [1]. In recent years, due to improvements in imaging and serological examination technology, including computed tomography, ultrasound, and alpha-fetoprotein (AFP) and des-gamma-carboxy prothrombin assays, the detection rate of early-stage HCC has increased. However, the mortality rate of HCC in China remains high, ranking as the second most lethal cancer in the country according to the GLOBCAN 2018 online database [2]. This is attributed the complex and heterogeneous nature of HCC, which involve multiple stages and factors. Although several tissue prognostic biomarkers, such as C-C motif chemokine ligand 14 (CCL14), cytokeratin 19 (CK19), CD133, and CD90, have been investigated, most of them can be located in the cytoplasm as well as on the cell membrane, which may lead to confusing results and nonspecific background staining. Therefore, substantial effort is still needed to identify novel biomarkers, especially nuclei-labeled markers, that can help improve the accuracy of prognosis predictions.

*EWSR1* (Ewing sarcoma breakpoint region 1/EWS RNA binding protein 1) was initially identified as a translocation-generated fusion gene between *EWSR1* and *FLI1* in Ewing’s sarcoma and neuroectodermal tumors [3]. Recently, *EWSR1* has been considered as a ‘hybrid’ gene involved in multiple mesenchymal tumor translocations, with evidence showing that it could be translocated and fused with many partner genes, including *EWSR1-FLI1* and *EWSR1-ERG* in Ewing’s sarcoma [4,5], *EWSR1-WT1* in desmoplastic small round cell tumors [6], *EWSR1-DDIT3* in myxoid liposarcoma [7], *EWSR1-CREB* in angiomatoid fibrous histiocytoma [8] and *EWSR1-ATF1* in clear-cell sarcoma-like tumors of the gastrointestinal tract. In addition, EWSR1 acts as a multifunctional RNA/DNA binding protein and is involved in various cellular processes, such as transcription regulation and RNA splicing [9]. Nevertheless, few studies have focused on the biological role of EWSR1 in epithelial tumors, especially in HCC.

The aim of this study was to investigate the expression and clinical significance of EWSR1 in HCC based on publicly available data and our cohorts. The biological function and pathway of the enrichments were explored through bioinformatics analysis, thereby providing valuable information on whether EWSR1 could be used as a candidate predictor of HCC clinical outcomes and an effective biomarker in routine clinical practice.

## Materials and methods

### TCGA and GEO datasets

*EWSR1* RNA-sequencing and detailed clinicopathological data profiles in HCC (n = 371) and healthy liver tissues (n = 50) from 1995 to 2015 were obtained from The Cancer Genome Atlas (TCGA) database (https://portal.gdc.cancer.gov/projects/TCGA-LIHC) [10]. Clinicopathological parameters were obtained for 369 cases, and a few of parameters with incomplete information were marked as Not reported. Gene expression profiles were obtained using R version 3.6.1 software (R Foundation for Statistical Computing, Vienna, Austria) from the E-MTAB-6695 cohort from October 2006 to November 2015, comprising seven Gene Expression Omnibus (GEO) datasets (accession no. GSE40873, GSE41804, GSE45436, GSE6222, GSE62232, GSE75271, and GSE29721) [11].

### Patients and tissue samples

A total of 124 paraffin-embedded HCC tissue specimens and adjacent healthy liver tissues, collected in 2013–2019 from patients naïve to preoperative radiotherapy or chemotherapy, were obtained from the Department of Pathology of The People’s Hospital of Jianyang City, China. The clinicopathological characteristics of all patients were obtained and the pathological diagnosis of each tissue specimen was confirmed by at least two pathologists. Follow-up data were acquired from the medical records and by telephone calls. Tumor differentiation was evaluated according to the World Health Organization Classification of Tumors of the Digestive System (5^th^ Edition) [12]. Disease-free survival (DFS) was measured from the date of surgery until disease relapse or metastasis, and overall survival (OS) was measured from the date of surgery until patient death. All patients provided written informed consent during hospitalization, and the Ethics Committee of The People’s Hospital of Jianyang City approved the study (No.2021017).

### Immunohistochemistry

Formalin-fixed and paraffin-embedded tissue sections (4 μm) were deparaffinized in xylene and rehydrated by serial ethanol washes. The slides were treated with 3% H_2_O_2_ for 15 min to quench the endogenous peroxidase. Antigen retrieval was performed by incubating the slides with 0.01 M citrate buffer (pH 6.0) at 100°C for 10 min. Standard immunohistochemical protocol was then implemented using a Ventana Benchmark XT autostainer (Ventana Medical Systems, Tucson, AZ, USA) as described previously [13]. Negative control slides omitting the primary antibodies were used for all assays.

### Evaluation and scoring

A semi-quantitative scoring system considering both intensity of staining and the extent of tumor cells was applied for EWSR1 (nuclear staining) and AFP (cytoplasmic staining). The staining intensity was scored as follows: 0, negative; 1, weak positive; 2, intermediate positive; and 3, strong positive. The scores of the extent of immunostaining signals were assessed according to the percentage of HCC cells that showed positive staining in each microscopic field (0–100). A final score ranging from 5 to 300, with a median score of 100, was achieved by multiplying the scores of intensity and extent. All cases were divided into two groups: low (score 0 − 100) and high (100 − 300) expression [14,15]. Ki67 status was evaluated based on the percentage of positive nuclear-stained tumor cells: low expression (≤ 25%) and high expression (> 25%) [16].

### PPI and KEGG/GO biological process enrichment

Correlation analysis was performed using the Pearson correlation test. Differentially expressed genes (DEGs) in protein-protein interaction analysis (PPI) with a connectivity degree > 20 and with a combination score > 0.75 were represented using Cytoscape (https://cytoscape.org) [17]. Gene ontology (GO) biological process [18] and Kyoto Encyclopedia of Genes and Genomes (KEGG) pathway enrichment analyses [19] were conducted.

### Statistical analysis

A chi-square test was applied to evaluate associations between the immunoreactivity of EWSR1 and other markers, as well as with the clinicopathological characteristics of HCC patients. Kaplan-Meier survival analysis was performed to estimate the prognostic relevance of EWSR1, and the survival difference between groups was assessed using the log-rank test. Univariate and multivariate Cox regression analyses were performed to evaluate the differences in all possible risk factors for death. SPSS 22.0 software (IBM Corp., Armonk, NY, USA) was used for all statistical analyses. For all tests, *P* < 0.05 was considered to indicate statistical significance.

## Results

In this study we explored the prognostic potential and role of EWSR1 in HCC. Based on the publicly available data, as well as in primary tissue samples, we demonstrate that EWSR1 is overexpressed in HCC, and is associated with high histological tumor grade and short overall patient survival. Moreover, EWSR1 was found to be an independent factor for poor prognosis in patients with HCC by univariate and multivariate cox regression analyses. GO and KEGG enrichments results also showed that EWSR1 was most likely to contribute for RNA splicing and DNA replication processes in HCC. These results, taken together, suggest that the EWSR1 may represent a valuable prognostic marker for HCC outcome in the clinical practice.

### EWSR1 is upregulated in HCC patients

**Figure 1.**
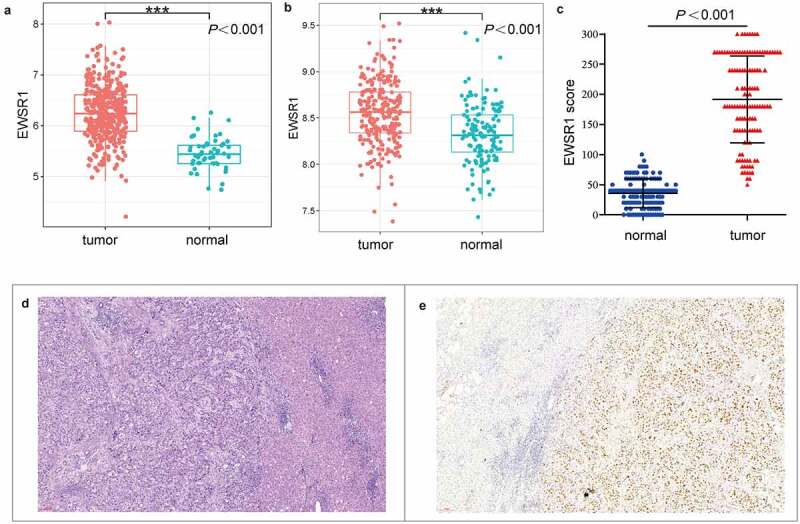
*EWSR1* expression between tumor and non-tumor liver tissues in HCC patients in TCGA (a) and GEO (b) datasets, and our cohort (c). Hematoxylin and eosin staining of HCC (left) and paratumoral, healthy (right) tissues (d). Positive immunohistochemical staining of EWSR1 in HCC (right), negative in paratumoral normal (left) tissue (e). Scale: 100 μm. Abbreviations: GEO, Gene Expression Omnibus; HCC, hepatocellular carcinoma; TCGA, The Cancer Genome Atlas

In this study, we first analyzed ESWR1 expression in the TCGA and E-MTAB-6695 cohorts. As shown in Figure 1, *EWSR1* levels were significantly upregulated in HCC tissues compared with healthy liver tissues (*P* < 0.001, Figure 1(a, b)). In addition, immunohistochemical analysis of EWSR1 in our cohort showed low expression in all the healthy liver samples (124/124) whereas it was overexpressed in 81.45% (101/124) of the HCC tissues, suggesting that EWSR1 is upregulated in HCC (*P*< 0.001, Figure 1(c)), which is consistent with data from TCGA and GEO databases.

### Overexpression of EWSR1 correlates with clinicopathological features of HCC

To explore the role of upregulated EWSR1 in HCC progression, the association between EWSR1 expression and patient clinicopathological features was evaluated using data from the TCGA database and our cohort. Analysis of TCGA data showed that overexpression of EWSR1 was associated with higher histological grade (*P* < 0.001), pathologic T stage (*P* = 0.026) and mortality (*P* = 0.007) (Table 1). In our cohort, the degree of tumor differentiation was lower in patients with high expression of EWSR1 as compared with those in the EWSR1 low-expression group (Table 1). Moreover, the Tumor (T) staging (*P* = 0.004), Nodule (N) staging (*P* = 0.008), and Tumor-Nodule-Metastasis (TNM) staging (*P* = 0.001), was well as AFP (*P* < 0.001) and Ki67 (*P* < 0.001) expression rates, and mortality (*P* < 0.001) were markedly higher in the EWSR1 overexpression group (Table 1). No significant differences in sex, age, tumor size, capsular invasion, vascular tumor thrombus, cirrhosis, recurrence rate, or other indicators were observed between the two groups (Table 2).

**Table 1.** Correlation between *EWSR1* expression and clinicopathological parameters of HCC patients in the TCGA cohort

Characteristics	EWSR1 expression		*P*
	High (n = 185)	Low (n = 184)	
Histologic grade, n (%)			
G1	17 (9.2)	38 (20.7)	< 0.001
G2	82 (44.3)	95 (51.6)	
G3	77 (41.7)	44 (23.9)	
G4	6 (3.2)	5 (2.7)	
Not reported	3(1.6)	2(1.1)	
Pathologic_M, n (%)			
M0	133 (71.9)	131 (71.2)	0.989
M1	2 (1.1)	2 (1.1)	
MX	50 (27.0)	51 (27.7)	
Pathologic_N, n (%)			
N0	128 (69.2)	122 (66.3)	0.426
N1	3 (1.6)	1 (0.5)	
NX	54 (29.2)	61 (33.2)	
Pathologic_T, (%)			
T1	78 (42.2)	101 (54.9)	0.026
T2	50 (27.0)	44 (23.9)	
T3	49 (26.5)	31 (16.9)	
T4	8 (4.3)	5 (2.7)	
TX	0 (0.0)	3(1.6)	
Days to death, mean (SD)	560.73 (609.94)	813.12 (753.01)	0.039
Sex, n (%)			
Female	65 (35.1)	55 (29.9)	0.335
Male	120 (64.9)	129 (70.1)	
Race, n (%)			
American Indian or Alaska native	1 (0.5)	1 (0.5)	0.377
Asian	83 (44.9)	74 (40.2)	
Black or African American	9 (4.9)	8 (4.4)	
Not reported	2 (1.1)	8 (4.4)	
White	90 (48.6)	93 (50.5)	
Vita status, n (%)			
Alive	107 (57.8)	133 (72.3)	0.007
Dead	78 (42.2)	50 (27.2)	
Not Reported	0 (0.0)	1 (0.5)	
Age, mean (SD)	58.94 (13.49)	60.57 (13.30)	0.246
Tumor stage, n (%)			
Not reported	13 (7.0)	11 (6.0)	0.068
Stage I	73 (39.5)	96 (52.2)	
Stage II	44 (23.8)	42 (22.8)	
Stage III	53 (28.6)	32 (17.4)	
Stage IV	2 (1.1)	3 (1.6)	

Abbreviations: HCC, Hepatocellular carcinoma; M, metastasis; N, nodule; T, tumor; TCGA, The Cancer Genome Atlas.

**Table 2.** Correlation between EWSR1 expression and clinicopathological parameters of HCC patients in our cohort

**Characteristics n (%)**	**EWSR1 expression**		**χ^2^**	***P***
	**Low (n = 64)**	**High (n = 60)**		
Sex			0.042	0.838
Male	58 (90.6)	55 (91.7)		
Female	6 (9.4)	5 (8.3)		
Age, mean (SD)			1.054	0.305
≤ 50 years	15 (23.4)	19 (31.7)		
> 50 years	49 (76.6)	41 (68.3)		
Tumor size			2.939	0.230
≤ 2 cm	6 (9.4)	3 (5.0)		
2–5 cm	28 (43.8)	20 (33.3)		
> 5 cm	30 (46.9)	37 (61.7)		
Capsule invasion			1.472	0.225
No	7 (10.9)	3 (5.0)		
Yes	57 (89.1)	57 (95.0)		
Lymphovascular infiltration			2.160	0.142
No	11 (17.2)	5 (8.3)		
Yes	53 (82.8)	55 (91.7)		
Differentiation degree			7.319	0.026
Poor	5 (7.8)	15 (25.0)		
Moderate	56 (87.5)	44 (73.3)		
Good	3 (4.7)	1 (1.7)		
Cirrhosis			3.228	0.072
No	14 (21.9)	6 (10.0)		
Yes	50 (78.1)	54 (90.0)		
T stage			13.327	0.004
T1	3 (4.7)	2 (3.3)		
T2	30 (46.9)	15 (25.0)		
T3	31 (48.4)	35 (58.4)		
T4	0 (0)	8 (13.3)		
N stage			6.991	0.008
N0	57 (89.1)	42 (70.0)		
N1	7 (10.9)	18 (30.0)		
TNM stage			17.348	0.001
I	3 (4.7)	2 (3.3)		
II	28 (43.7)	9 (15.0)		
III	30 (46.9)	35 (58.4)		
IV	3 (4.7)	14 (23.3)		
AFP expression			42.701	< 0.001
Low	47 (73.4)	9 (15.0)		
High	17 (26.6)	51 (85.0)		
Ki67 expression			16.814	< 0.001
Low	37 (57.8)	13 (21.7)		
High	27 (42.2)	47 (78.3)		
Recurrence			0.176	0.675
No	16 (25.0)	17 (28.3)		
Yes	48 (75.0)	43 (71.7)		
Death			22.642	< 0.001
No	41 (64.1)	13 (21.7)		
Yes	23 (35.9)	47 (78.3)		

Abbreviations: AFP, alpha-fetoprotein; HCC, Hepatocellular carcinoma; N, nodule; T, tumor; TNM; tumor-nodule-metastasis.

### Upregulation of EWSR1 correlates with poor prognosis in HCC

**Figure 2.**
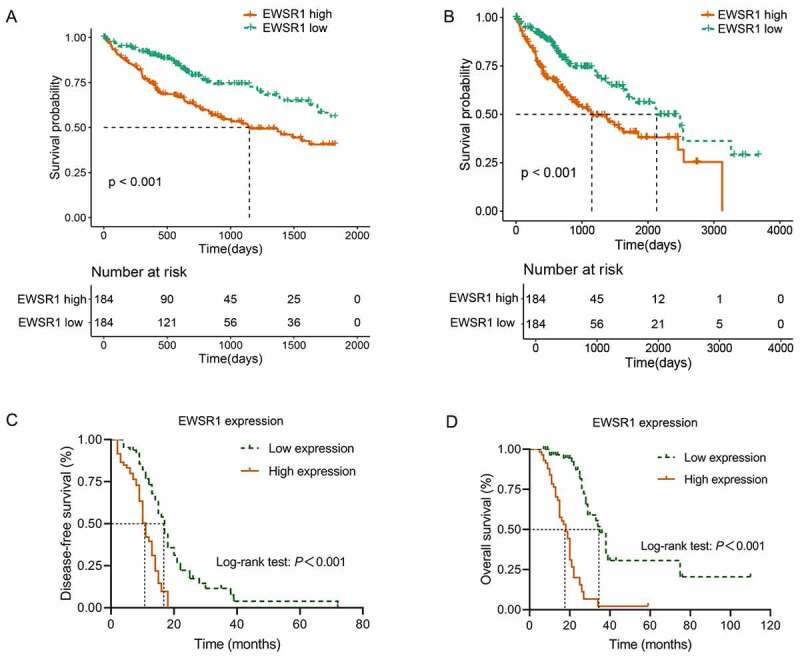
Comparison of the 5- and 10-year overall survival between EWSR1 high and low groups in The Cancer Genome Atlas (TCGA) dataset (a, b) and our cohort (c, d)

To evaluate the prognostic significance of EWSR1, we examined the association between EWSR1 expression and OS in HCC patients. Kaplan-Meier analyses indicated that overexpression of EWSR1 was associated with poor OS (*P* < 0.01, Figure 2(a,b)) in the TCGA dataset, and the DFS and OS curve of our cohort also showed that HCC patients with low EWSR1 expression had better clinical outcomes than those with high expression (*P* < 0.01, Figure 2(c,d)). We then performed a Cox regression model to evaluate the independent risk factors for disease-free survival (DFS) and OS based on our cohort. The mean DFS and OS were of 17 (95% confidence interval [CI]: 14.78–19.23) and 35 months (95% CI: 30.74–39.26), respectively, in the EWSR1 low expression group; and 11 (95% CI: 9.76–12.24) and 18 months (95% CI: 14.20–21.80), respectively, in the EWSR1 high expression group. Univariate Cox regression analysis showed that T stage, N stage, TNM stage, AFP overexpression, and EWSR1 overexpression were significantly associated with DFS (*P* < 0.05). Multivariate Cox regression analysis further confirmed that AFP and EWSR1 overexpression were independent risk factors for DFS (*P* < 0.05, Table 3). In addition, univariate Cox regression analysis showed that tumor size, differentiation degree, T stage, N stage, TNM stage, AFP overexpression, Ki67 expression, and EWSR1 overexpression were each significantly associated with OS (*P* < 0.05). Following multivariate analysis confirmed that T stage, N stage, AFP and EWSR1 overexpression were independent risk factors for OS (*P* < 0.05, Table 4).

**Table 3.** Univariate and multivariate Cox regression analyses of DFS in HCC patients

Parameter	Univariate analysis		Multivariate analysis	
	HR (95% CI)	*P*	HR (95% CI)	*P*
Gender (vs. male)	1.257 (0.544–2.901)	0.592		
Age	0.673 (0.430–1.053)	0.083		
Tumor size	1.348 (0.972–1.869)	0.073		
Capsule invasion	1.880 (0.809–4.366)	0.142		
Lymphovascular infiltration	1.662 (0.852–3.240)	0.136		
Differentiation degree	0.962 (0.576–1.605)	0.882		
Cirrhosis	1.710 (0.927–3.153)	0.086		
T stage	1.413 (1.016–1.966)	0.040	1.184 (0.682–2.055)	0.549
N stage	2.226 (1.303–3.800)	0.003	1.498 (0.664–3.379)	0.330
TNM stage	1.542 (1.142–2.081)	0.005	1.121 (0.660–1.904)	0.672
AFP	3.133 (1.962–5.002)	< 0.001	2.339 (1.330–4.111)	0.003
Ki67	1.417 (0.921–2.181)	0.113		
EWSR1 overexpression	3.177 (1.947–5.185)	< 0.001	1.654 (1.098–3.011)	0.041

Abbreviations: AFP, alpha-fetoprotein; CI, coefficient interval; DFS, disease-free survival; HCC, Hepatocellular carcinoma; HR, hazard ratio; N, nodule; T, tumor; TNM; tumor-nodule-metastasis.

**Table 4.** Univariate and multivariate Cox regression analyses of OS in HCC patients

Parameter	Univariate analysis		Multivariate analysis	
	HR (95% CI)	*P*	HR (95% CI)	*P*
Gender (vs. male)	3.513 (1.325–9.315)	0.012		
Age	0.938 (0.566–1.556)	0.805		
Tumor size	1.526 (1.017–2.290)	0.041	0.769 (0.391–1.513)	0.447
Capsule invasion	4.089 (0.995–16.808)	0.051		
Lymphovascular Infiltration	2.306 (0.990–5.369)	0.053		
Differentiation degree	0.454 (0.275–0.750)	0.002	0.987 (0.525–1.856)	0.967
Cirrhosis	1.748 (0.888–3.439)	0.106		
T stage	2.276 (1.479–3.502)	< 0.001	2.887 (1.374–6.065)	0.005
N stage	4.043 (2.269–7.202)	< 0.001	3.626 (1.510–8.706)	0.004
TNM stage	2.970 (1.972–4.473)	< 0.001	1.145 (0.644–2.036)	0.645
AFP	5.825 (3.184–10.654)	< 0.001	2.715 (1.337–5.516)	0.006
Ki67	1.753 (1.055–2.911)	0.030	0.932 (0.526–1.652)	0.811
EWSR1 overexpression	5.914 (3.472–10.074)	< 0.001	3.492 (1.824–6.685)	< 0.001

Abbreviations: AFP, alpha-fetoprotein; CI, coefficient interval; HCC, Hepatocellular carcinoma; HR, hazard ratio; N, nodule; OS, overall survival; T, tumor; TNM; tumor-nodule-metastasis.

### Bioinformatic analysis of EWSR1 in HCC

**Figure 3.**
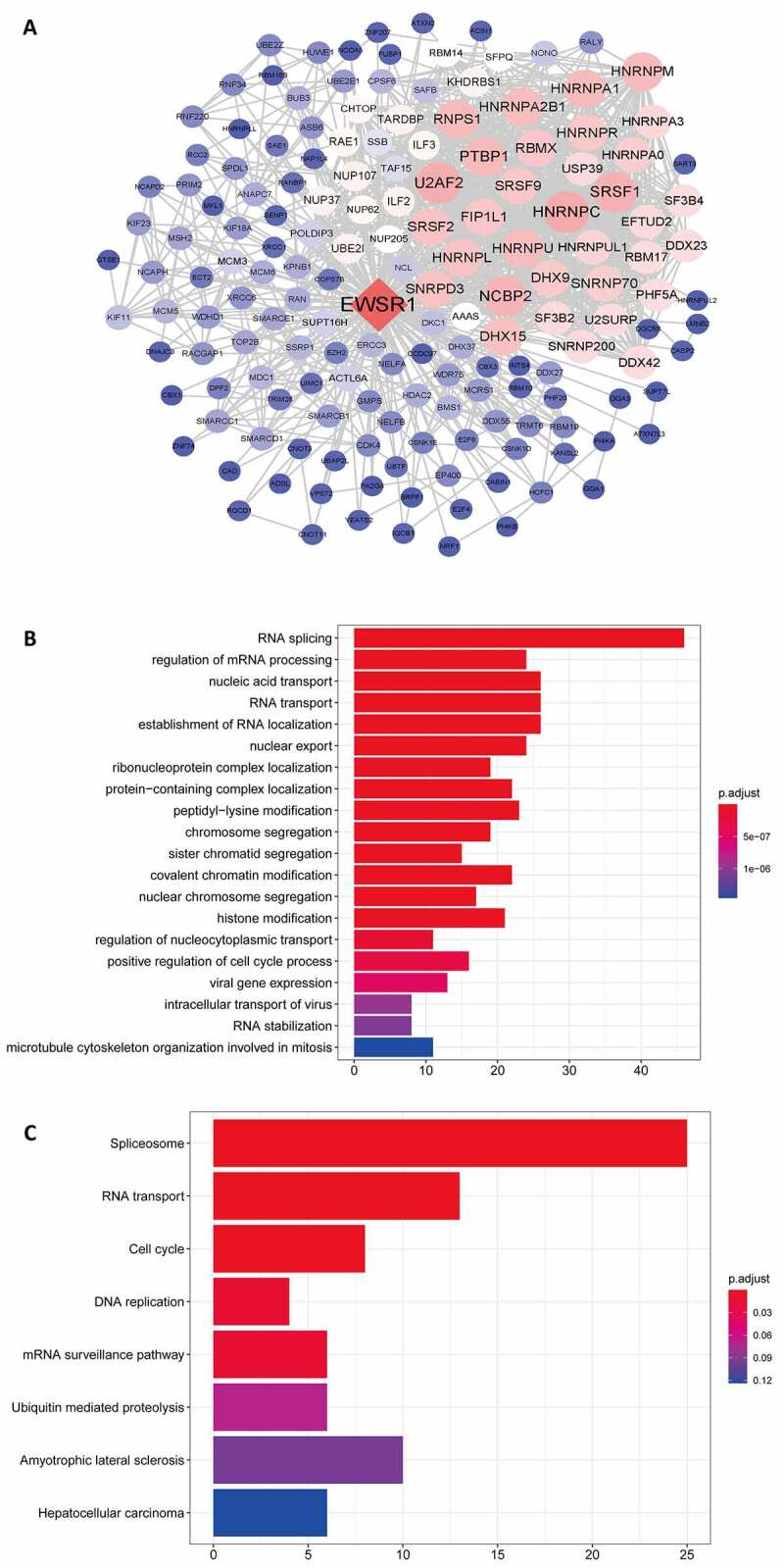
Protein-protein interaction network of *EWSR1* (a). Gene ontology (GO) (b) and Kyoto Encyclopedia of Genes and Genomes (KEGG) pathway (c) analyses of coexpressed genes of *EWSR1.*

Using data from TCGA, we performed the Pearson correlation test on *EWSR1* and other differential genes (with a threshold of 0.7), and defined the PPI network of *EWSR1* using Cytospace (Figure 3(a)). Subsequently, the identified genes of the PPI with adjusted *P* < 0.05 were further analyzed by GO and KEGG pathway analyses. GO functional enrichment analysis revealed significantly enriched biological processes, including RNA splicing, mRNA processing, nucleic acid and RNA transport, and establishment of RNA localization and nuclear export (Figure 3(b)). KEGG pathway analysis further showed that the spliceosome, RNA transport, cell cycle, and DNA replication were the most enriched (Figure 3(c)), suggesting that EWSR1 plays a significant role in the RNA splicing and DNA replication processes.

## Discussion

The EWSR1 gene is a member of the TET (also known as FET) family [20], including Fused in sarcoma/Translocated in Liposarcoma (FUS/TLS) and TATA-box binding Associated Factor 15 (TAF15) [21]. As mentioned before, *EWSR1* is well-known to be able to fuse with a range of genes in the mesenchymal neoplasms, and a few series of epithelial tumors as myoepithelial[22], primary clear cell carcinoma of the thymus [23] and the Xp11 translocation renal cell carcinoma [24]. Recently, *EWSR1* rearrangement is reported as a frequent event in papillary microcarcinoma and related to classic and small-cell morphology [25]. More than that, EWSR1 can participate in a variety of cell processes by regulating gene expression, mitotic cell division, RNA splicing, cell signaling and DNA repair and transcription [9]. However, the role of EWSR1 protein expression in tumor, especially in epithelial cancers is rarely reported.

In the present study, *EWSR1* was found to be significantly upregulated in HCC tissues compared with the healthy counterparts, as determined by the bioinformatic analysis of GEO and TCGA datasets, was well by immunohistochemical assessment in primary samples. Hence, EWSR1 may be considered a novel biomarker to distinguish HCC from healthy liver. Noteworthy, the expression of EWSR1 was found to be closely related to aggressive factors and mortality, further illustrating that EWSR1 expression is positively correlated with poor tumor differentiation and advanced clinical stage. EWSR1 overexpression was reported to be a poor prognostic predictor in multiple myeloma [26], however, to date, no investigations on the role of EWSR1 in HCC were available. Analysis of high-throughput RNA sequencing data publicly available showed that increased *EWSR1* expression was associated with shortened OS after liver resection in this work, and this observation was further confirmed in our cohort, in which EWSR1 overexpression was correlated with significantly shorter PFS and OS after a 5-year follow-up period. In addition, analysis using the Cox proportional hazards regression models showed that overexpression of EWSR1 is an independent predictor of PFS and OS in HCC patients. Therefore, these findings support the use of EWSR1 as a novel and valuable biomarker for predicting poor prognosis in HCC.

To our knowledge, several prognostic molecular markers for HCC have been revealed in the recent years. In Gu’s study, CCL14 is not only a potential prognostic biomarker but also correlated with tumor immune cells infiltration in HCC [27]. CK19, a traditional bile duct marker, has been increasingly reported to be associated with aggressive behaviors and poor outcomes in HCC. Furthermore, as TGFβR1 may be a targeted therapeutic factor for CK19 positive HCC, it is supported to include CK19 positive hepatocellular carcinoma as a special subtype [28]. Ma et al reveal Collagen 1A1 (COL1A1) can be used as a survival advantage predicator and downregulation of COL1A1 could suppress the oncogenicity and epithelial-to-mesenchymal transition process of HCC cells [29]. AFP, a serum marker for HCC detection, is also frequently elevated in HCC, recenty study show AFP acetylation can promote HCC progress by blocking binding to the phosphatase PTEN and the pro-apoptotic protein caspase-3 [30]. In our samples, AFP was overexpressed in HCC tissues and acted as an independent prognostic factor. However, due to the high heterogeneity of HCC, a panel of markers should be used to comprehensively evaluate the prognosis assessment. As most of the markers reported are located in the cytoplasm or on the cell membrane, which may lead to confusing results and nonspecific background staining. EWSR1, as a marker for nuclear staining, has the unique advantage of being able to evaluate results more accurately and reduce false negatives and false positives. Comparison of specificity and sensitivity of EWSR1 with other markers will be carried out in the future.

In the past decades, studies on EWSR1 have mainly focused on the role of fusion proteins formed by *EWSR1* translocation in tumorigenesis and cancer development [31], whereas the biological function of EWSR1 itself remains poorly understood. EWSR1 is known to be involved in mitotic progression by promoting microtubule acetylation in the mitotic spindle and inhibiting the activity of HDAC6 [32]. We performed a comprehensive bioinformatics analysis of the TCGA dataset to reveal the underlying mechanism of EWSR1 in HCC. In accordance with the previously reported role of EWSR1, herein we found that *EWSR1* and is related/partner genes were mainly involved in RNA splicing, mRNA processing, nucleic acid transport, and RNA transport processes. Moreover, the spliceosome was the most enriched pathway, as determined by KEGG pathway analysis. Taken together, these results confirm that *EWSR1* may contribute for HCC pathological mechanisms by partaking in RNA splicing and DNA replication.

## Conclusion

To our knowledge, this is the first study to investigate the biological function of EWSR1 in an epithelial cancer. Our results suggest that high EWSR1 expression is an independent predictor of shorter DFS and OS in patients with HCC, by regulating RNA splicing and DNA replication. Taken together, these results indicate that EWSR1 may represent as a novel biomarker of poor prognosis in patients with HCC.

## Research highlights

- EWSR1 expression is upregulated in HCC tissues compared with paratumor tissues.
- EWSR1 overexpression is associated with high histological grade and short OS.
- EWSR1 is an independent factor for poor prognosis in patients with HCC.
- RNA splicing processing represents the major function and pathway of EWSR1 in HCC.
